# Recurrent complex spinal tuberculosis accompanied by sinus tract formation: causes of recurrence and clinical treatments

**DOI:** 10.1038/s41598-018-25142-z

**Published:** 2018-05-02

**Authors:** Biao Wang, Lingbo Kong, Ziqi Zhu, Wenjie Gao, Hua Guo, Xiaodong Wang, Hui Li, Qinpeng Zhao, Haiping Zhang, Dingjun Hao

**Affiliations:** Department of Spine Surgery, Honghui Hospital, Xi’an Jiaotong University College of Medicine, No. 76 Nanguo Road, Xi’an, 710054 Shanxi China

## Abstract

Recurrent complex spinal tuberculosis accompanied by sinus tract formation is one of the most common and complex types of recurrent tuberculosis. To date, only very few studies have reported the strategies and effectiveness of surgical treatments on postoperative recurrent spinal tuberculosis accompanied by sinus tract formation. In this study, 21 recurrent patients out of 87 patients with complex spinal tuberculosis were reviewed. The data of the patients, including age, gender, existence of drug-resistant mycobacterium tuberculosis, postoperative standard chemotherapy, completeness of lesion debridement, reconstruction of the spinal stability, nutritional status, accompaniment by tuberculosis in other regions, timing of the operations, and areas of the lesions, were collected for single- and multiple-factor analyses. The clinical outcomes were evaluated by long-term follow-ups. The results showed that 7 factors were significantly associated with postoperative recurrence of complex spinal tuberculosis accompanied by sinus tract formation. This suggested that when we treat complex spinal tuberculosis, we should pay more attention to these seven indicators to avoid postoperative recurrence, and our clinical outcomes suggested that it is difficult to treat recurrent complicated spinal tuberculosis combined with sinus tract formation. The key for successful surgical treatment includes effective chemotherapy, radical debridement and proper reconstruction of spinal stability.

## Introduction

Approximately 300 years ago, Percival Pott reported the first modern case of spinal tuberculosis in 1779^1^. Spinal tuberculosis, as one of most common types of extra pulmonary tuberculosis, accounts for 50–60% of osteoarticular tuberculosis cases all over the body and is one of the common causes of spinal kyphosis and neurological deterioration^2–4^. With the development of medical technologies and advances in the understanding of spinal tuberculosis, standard chemotherapy and aggressive surgical interventions have been accepted by numerous researchers^5–9^. However, due to the appearance of drug-resistant mycobacterium tuberculosis, non-standard chemotherapy and surgical treatments and to the fact that most patients cannot receive timely and appropriate treatments^10–12^, spinal tuberculosis tends to be complex and serious. The treatment procedures become more complex, while the clinical effectiveness is unclear^13^. In our treatment centre, the following criteria were used for the diagnosis of complex spinal tuberculosis: 1) erythrocyte sedimentation rate (ESR) that remains >100 mm/h 1 month after the start of standard anti-tuberculosis chemotherapy; 2) multi-segmental spinal tuberculosis that damaged 3 or more segments; 3) combination with tuberculosis of other organs; 4) combination with 2 or more large abscesses; 5) combination with sinus tract formation; 6) combination with neurological deterioration; and 7) postoperative recurrence of spinal tuberculosis. The patients were diagnosed with complex spinal tuberculosis if two or more of these conditions were found. Complex spinal tuberculosis is characterized by a long disease course, severe disease conditions, and complex and various clinical manifestations; in addition, the clinical treatments for this disease are generally tricky, and the postoperative recurrence rate is relatively high.

The earliest study reported that the postoperative recurrence rate of complex spinal tuberculosis was as high as 60%^14^. The manifestations of a postoperative recurrence of tuberculosis mainly include no substantial improvement in the systemic and local symptoms, enlargement of abscesses, unhealed incisions, and sinus tract formation, all of which severely affect the life quality of the patients. Among these symptoms, the combination with sinus tract formation is one of the most common and complex ones. To date, studies have mainly investigated the clinical effectiveness on spinal tuberculosis accompanied by sinus tract formation before initial surgical treatments. However, only a few studies have reported the strategy of surgical treatments and their effectiveness on postoperatively recurrent spinal tuberculosis accompanied by sinus tract formation.

In the present study, we retrospectively analysed the clinical data of 21 patients with recurrent tuberculosis accompanied by sinus tract formation after an initial operation, who then underwent sinus tract excision, lesion debridement, and bone graft fusion and internal fixation. The aims of this study were as follows: (1) to summarize the reasons for postoperative recurrence of spinal tuberculosis and sinus tract formation; and (2) to summarize the strategy and clinical prognosis of surgical treatments for postoperative recurrence of spinal tuberculosis accompanied by sinus tract formation.

## Patients and Methods

### Inclusion and exclusion criteria

The inclusion criteria of the patients were as follows: (1) present postoperative recurrent spinal tuberculosis with no response after 6 months of standard drug chemotherapy; (2) accompanied by sinus tract formation; (3) the lesion included sequestrum or an evident paravertebral abscess; and (4) bacterial culture of the sinus tract secretions suggested no mixed infection.

The exclusion criteria were as follows: (1) disease recovery after drug chemotherapy; (2) bacterial culture of the sinus tract secretions suggested mixed infection; and (3) combination with active pulmonary tuberculosis.

The criteria for diagnosing the recurrence of tuberculosis were as follows: the presence of persistent symptoms of tuberculosis poisoning; the appearance of a localized mass or cold abscess 6 months post-operation or formation of a sinus tract; and imaging examinations that confirmed the existence of sequestrum or an evident paravertebral abscess.

### General data

From January 1997 to January 2012, 663 patients with spinal tuberculosis were treated in our department, among whom were 87 patients with complex spinal tuberculosis and 21 patients with postoperative recurrent tuberculosis. The overall recurrence rate was 3.2%, and the recurrence rate of complex tuberculosis was 24.1%. Among the 66 patients who recovered, MRI scanning at 3 months after the operation showed the formation of paravertebral cold abscesses in 3 patients, but no sinus tract formation was found; the disease in these 3 patients improved gradually after bed rest, nutrition support, immobilization with a brace, and intensive multidrug chemotherapy, and these 3 patients recovered within the 6 months following the initial operation. Twenty-one patients exhibited postoperative recurrence, and all these cases were accompanied by sinus tract formation, and the disease protracted and did not recover. Among these 21 patients, there were 12 males and 9 females; the mean age of the patients was 38.8 ± 11.6 years (range: 22–58 years). The disease duration before the initial diagnosis was 12.8 ± 4.8 months (range: 6–23 months), and the time between the initial operation and the recurrence was 10.9 ± 3.8 months (range: 6–18 months). The locations of the lesions were as follows: 4 in the thoracic segment, 6 in the thoracolumbar segment; 10 in the lumbar segment, and 1 in the lumbosacral segment. The areas involved in the lesions were as follows: 2 patients with one vertebral body involved, 13 patients with two vertebral bodies involved, and 6 patients with three or more vertebral bodies involved.

Six cases were also accompanied by tuberculosis in other regions (with active pulmonary tuberculosis ruled out) before the initial operation (including 2 patients with tuberculosis of the shoulder joint, 2 patients with tuberculosis of the hip joint, and 2 patients with renal tuberculosis). The complications before the initial operation were as follows: 7 patients had diabetes, and 8 had hypertension. The mean ESR of the patients at the initial hospitalization was 96.4 ± 20.8 mm/h (range: 65–130 mm/h), and the mean ESR reduced to 67.6 ± 9.2 mm/h (range: 55–83 mm/h) before the initial operation (Table 1).

**Table 1:** Demographic and clinical characteristics of the recurrent patients.

Characteristics	Value
Number of patients	21
Age (yrs.)	38.8 ± 11.6 (range: 22–58)
Male to female ratio	12: 9
Disease duration before the initial diagnosis (months)	12.8 ± 4.8 (range: 6–23)
Time from initial operation to the recurrence (months)	10.9 ± 3.8 (range: 6–18)
Locations of the lesions	Thoracic: 4, Thoracolumbar: 6, Lumbar: 10, Lumbosacral: 1.
Accompanied by tuberculosis in other regions	6 patients
ESR at the initial hospitalization (mm/h)	96.4 ± 20.8 (range: 65–130)
ESR before the initial operation (mm/h)	67.6 ± 9.2 (range: 55–83)
Drug resistance	15 patients
Segments of the lesions	1 segment: 2 cases; 2 segments: 13 cases; ≥ 3 segments: 6 cases.

ESR: erythrocyte sedimentation rate.

Data are presented as the mean ± standard deviation.

The methods of the initial operation were as follows: 4 patients underwent anterior lesion debridement and bone graft fusion in one-stage operations; 8 patients underwent anterior lesion debridement, bone graft fusion and internal fixation in one-stage operations; 3 patients underwent anterior lesion debridement and bone graft fusion in one-stage operations and anterior internal fixation in two-stage operations; 4 patients underwent anterior lesion debridement and bone graft fusion and then posterior bone graft fusion and internal fixation in one-stage operations; and 2 patients underwent anterior lesion debridement and bone graft fusion in one-stage operations and posterior bone graft fusion and internal fixation in two-stage operations.

Sinus tract formation was observed in all patients at the incision made in the prior operation, and the number of sinus tracts was between 1 and 5. Mycobacterium cultures and drug sensitivity tests were performed in all the patients before the revision surgery, and the results showed that 15 patients demonstrated drug resistance. In brief, 7 patients were resistant to isoniazid, rifampicin, and streptomycin; 5 patients were resistant to isoniazid, rifampicin, streptomycin, and ethambutol; and 3 patients were resistant to isoniazid, rifampicin, streptomycin, and pyrazinamide. The regular bacterial cultures of the secretions from the sinus tract before the second operation all showed negative results. Nine patients were found to have neurological deterioration before the operation, and 1, 2, and 6 patients were classified with the American Spinal Injury Association’s (ASIA) grades B, C, and D, respectively.

### Imaging examinations

MRI and CT examinations showed that all the patients had evident residual sequestra at the site of the lesion, most of which were located on the side contralateral to the route of the lesion debridement. Ten of the patients had evident paravertebral abscesses. Non-fusion of the bone graft and pseudoarticulation formation was found in 5 patients, and progressive kyphotic deformity was found in 2 patients. The mean degree of the Cobb angle of the kyphosis was 9.0 ± 3.3° after the initial operation, and it was 19.1 ± 5.8° after the recurrence.

### Preoperative preparation

Considering that all the patients in this study had recurrent spinal tuberculosis, appropriate anti-tuberculosis drugs were chosen according to the results of the drug sensitivity tests before the operation, and levofloxacin (0.4 g/d) was used in combination with the chemotherapy to ensure that at least 4 effective chemotherapeutic drugs were used; the preoperative chemotherapy lasted for 3 months in order to control the symptoms of tuberculosis poisoning as much as possible. Regular clearing and dressing was performed for the local sinus tract to prevent mixed infection. The disease courses of the patients in this study were all relatively long; in addition, all the conditions of the patients were accompanied by sinus tract formation, heavy exudation, and low levels of haemoglobin and albumin. Therefore, small amounts of fresh erythrocyte suspensions and human albumin were infused at multiple times to correct the malnutrition.

### Operation methods

#### Principles of re-operation

The approach of the re-operation was determined according to the location of the sinus tract, area of the abscess, and location of the sequestrum. For the cases in which the abscess and sequestrum were mainly located on the side of the sinus tract, the entry point of the operation was selected to be the side of the sinus tract, while for the cases in which there was also evident abscesses and sequestra on the contralateral side, one-stage lesion debridement was performed through an approach on the contralateral side.

During the operation, the surgeons strictly abided by the principle of “asepsis”. The sinus tract was circularly resected from the surrounding normal tissues until the lesion of the vertebral body. We suggested that the normal tissues at least 0.5 cm around the sinus tract should be resected to ensure that the inflammatory tissues around the sinus tract were completely resected. The resected sinus tract tissues were categorized as the external part, middle part, and inner part and were then stored and used for the normal bacterial cultures, mycobacterium tuberculosis cultures, and drug sensitivity tests. Specific drug treatments were performed after the operation according to the test results.

The areas that were exposed should have been large enough to ensure that the lesions and the surrounding sclerotic bone were exposed sufficiently. For the patients who had an internal fixation during the initial operation, the exposure must have been sufficient, and the internal fixation was removed; the operation field was repeatedly rinsed with hydrogen peroxide and normal saline, and then the bone graft was implanted during the initial operation. The sequestra and surrounding sclerotic bone were resected successively until normal bone tissue with errhysis was revealed. An appropriately sized iliac bone autograft was implanted at the site of the bone defect.

For the patients with evident paravertebral abscesses, each abscess was incised according to the areas shown on the images, and then the abscess was removed under direct vision. Inserting a drainage tube into the abscess for the lesion debridement, which is the conventional method that is used, should be avoid, as this method could result in a dead cavity with residual abscesses. Saline-soaked gauze was used for the curettage of the abscess wall until fresh errhysis appeared, in order to ensure that the abscess tissue was completely removed. For the patients in whom an abscess on the contralateral side was also evident, an operation using the contralateral approach was performed to remove the abscess.

The method of the internal fixations was determined according to the abscess around the internal fixation during the initial operation and the local stability of the spine. For the patients in whom the abscess was not obvious but the sequestrum in the lesion area was obvious, and imaging examinations suggested poor local stability, more segments were fixed. For the patients with obvious abscesses, we suggested that the anterior internal fixation should be removed, and then the internal fixation with a pedicle screw-rod system was performed using the posterior approach. For the patients in whom the fixation in the initial operation was performed using the posterior approach and the lesion involved the internal fixation, the internal fixation was removed using the posterior approach, and then the internal fixation was performed again, with more segments fixed also according to the posterior approach.

#### Surgical Methods

**Figure 1:**
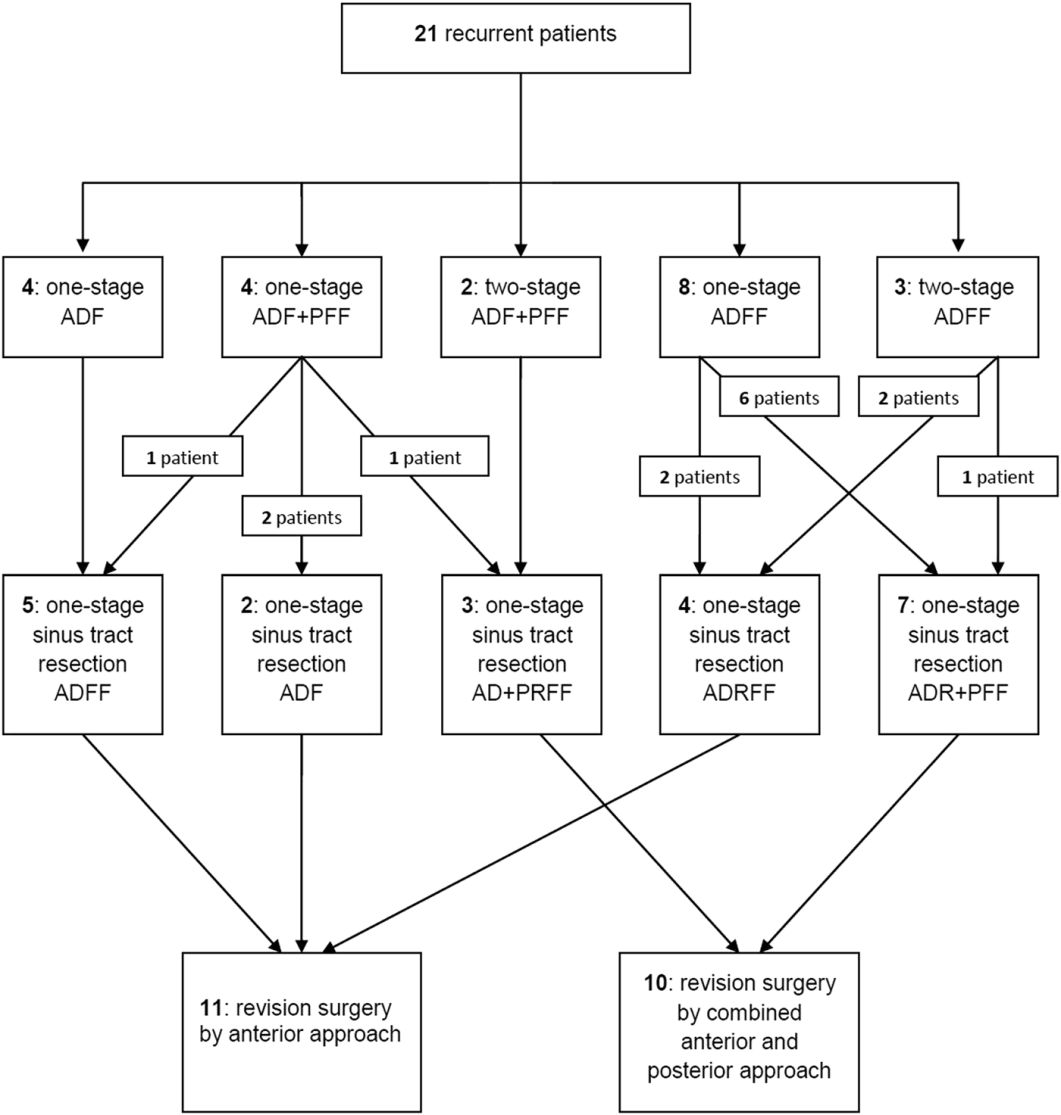
A schematic drawing of the elements of the revision surgery strategy. (AD: anterior debridement; ADF: anterior debridement and fusion; ADFF: anterior debridement, fusion and fixation; ADR: anterior debridement and internal fixation removal; ADRFF: anterior debridement, internal fixation removal, fusion and fixation; PFF: posterior fusion and fixation; PRFF: posterior internal fixation removal, fusion and fixation).

Four patients in this study underwent lesion debridement and bone graft fusion using the anterior approach in the initial operation; therefore, the sinus tract resection, lesion debridement, bone graft fusion and internal fixation were performed in the revision operation. In 1 patient, the initial operation involved anterior lesion debridement, a posterior bone graft fusion and internal fixation; due to an abscess that was not obvious, the anterior sinus tract resection, lesion debridement, bone graft fusion and internal fixation were performed in a re-operation. Four patients underwent one-stage anterior sinus tract resections, lesion debridement, internal fixation removal, bone graft fusion and internal fixation. Seven patients underwent one-stage anterior sinus tract resections, lesion debridement, internal fixation removal, and then posterior bone graft fusion and internal fixation were performed in a revision operation. Two patients underwent anterior lesion debridement, posterior bone graft fusion and internal fixation during the initial operation; the spinal posterior bone graft was well fused in these two patients, and the recurrent lesion did not involve the internal fixation system; therefore, the anterior sinus tract resection, lesion debridement, and bone graft fusion were performed in a re-operation. Three patients were found to have recurrent lesions involving the posterior pedicle screw internal fixation system; therefore, the anterior sinus tract resection, lesion debridement, posterior internal fixation removal, bone graft fusion and internal fixation were performed in a re-operation. A schematic diagram of the revision surgery strategy is shown in Fig. 1.

### Postoperative treatment

Lesion tissue was used for mycobacterium tuberculosis cultures and for drug sensitivity tests that were performed again after the operation, and then appropriate anti-tuberculosis drugs were selected according to the results of the drug sensitivity tests and drug resistance assays, which were used in combination with levofloxacin (0.4 g/d) to ensure that at least 4 types of effective chemotherapy drugs were used for the anti-tuberculosis therapy. The duration of the therapy was 18–24 months. Complications during the chemotherapy were closely monitored, and the strategy of chemotherapy was adjusted immediately once any complications occurred.

### Follow-up and effectiveness assessment

The patients were followed up with by the outpatient department at postoperative months 1, 3, 6, 9 and 12 and then were followed up with once semi-annually. At each follow-up, imaging examinations (X-ray, CT, and MR), liver and renal functions, and ESR were measured. The recovery criteria for spinal tuberculosis were as follows^6^: no recurrent tuberculous lesions after the 6-month follow-up; ESRs in the normal range; X-rays showing bony union of the lesion; and the patient maintained normal activities and work for 3–6 months. The recovery of neurological functions was assessed with the ASIA grades.

### Factors associated with the recurrence of tuberculosis

In total, 21 patients were included in the recurrent group, and 66 patients were included in the recovery group. Ten datasets of the patients, which included age, gender, existence of drug-resistant mycobacterium tuberculosis, postoperative standard chemotherapy, completeness of the lesion debridement, reconstruction of spinal stability, nutritional status, accompaniment with tuberculosis in other organs, timing of the operation, and segments of the lesion, were collected by reviewing the patients’ medical records. The detailed methods and results of the quantization and valuation of the data in both the recurrent and recovery groups are shown in Table 2.

**Table 2:** The results of the quantization and valuation of the data in the recurrent and recovery groups.

Factor	Recurrent group (n = 21)	Recovery group (n = 66)
Gender	Male (12), female (9)	Male (41), female (25)
Age (yrs.)	<30(6), 30–40(5), >40(10)	<30(18), 30–40(16), >40(32)
Existence of drug-resistant mycobacterium tuberculosis	Resistance(15), Non-resistance(6)	Resistance(11), Non-resistance(55)
Postoperative standard chemotherapy	Standard(4), non-standard(17)	Standard(8), non-standard(58)
Completeness of lesion debridement	Complete(9), non-complete(12)	Complete(48), non-complete(18)
Reconstruction of the spinal stability	Stabilized(17), non-stabilized(4)	Stabilized(60), non-stabilized(6)
Nutritional status	Normal(0), Moderate malnutrition(11), Severe malnutrition(10)	Normal(21), Moderate malnutrition(31), Severe malnutrition(14)
Accompaniment by tuberculosis in other organs	Yes(6), no(15)	Yes(8), no(58)
Timing of operation	ESR < 60 mm/h(4), ESR ≥ 60 mm/h(17)	ESR < 60 mm/h(18), ESR ≥ 60 mm/h(48)
Segments of lesion	Segments <3(2),segments ≥3(19)	Segments <3(8),segments ≥3(58)

### Statistical analysis

The SPSS 19.0 software (SPSS Company, USA) was used for the statistical analyses. Logistic regression was used for the single- and multiple-factor analyses, and the optimal variables were selected using the stepwise method. P < 0.05 was considered statistically significant.

### Ethics

We confirm that all methods were carried out in accordance with relevant guidelines and regulations, and all experimental protocols were approved by Honghui Hospital Xi’an Jiaotong University College of Medicine. We confirm that informed consent was obtained from all subjects.

## Results

All the patients were followed up with, and the mean follow-up time was 4.2 ± 0.8 years (3–6 years). The sinus tract tissues were collected during the operation, and bacterial cultures showed no infection by other bacteria in addition to the mycobacterium tuberculosis.

### Reasons for tuberculosis recurrence

The single-factor analysis showed that 7 factors, including the existence of drug-resistant mycobacterium tuberculosis, standard postoperative chemotherapy, completeness of the lesion debridement, reconstruction of spinal stability, nutritional status, accompaniment by tuberculosis in other regions, and the timing of the operation, were significantly associated with postoperative recurrence of complex spinal tuberculosis accompanied by sinus tract formation (*P* < 0.05), while the other factors were not significantly associated with recurrence (Table 3). The multi-factor analysis also showed that these 7 factors were significantly associated with postoperative recurrence of complex spinal tuberculosis accompanied by sinus tract formation (*P* < 0.05) (Table 4).

**Table 3:** The single factor Logistic regression analysis results of the reasons for recurrent complex spinal tuberculosis accompanied with sinus tract formation.

Risk factor	Regression coefficients	Standard errors	Chi-square value	Degree of freedom	*p*-value	95% confidence interval	
						Lower	Upper
Gender	0.121	0.531	0.059	1	0.823	0.410	3.211
Age (yrs.)	0.281	0.279	1.488	1	0.314	0.819	2.311
Existence of drug-resistant mycobacterium tuberculosis	2.915	0.521	27.455	1	0.000	6.112	40.987
Postoperative standard chemotherapy	2.541	0.544	22.784	1	0.000	4.789	37.787
Completeness of lesion debridement	2.478	0.532	20.246	1	0.000	4.012	34.578
Reconstruction of the spinal stability	1.354	0.490	4.578	1	0.018	1.079	11.778
Nutritional status	1.641	0.569	7.968	1	0.002	1.675	16.898
Accompaniment by tuberculosis in other organs	1.578	0.543	8.010	1	0.004	1.578	13.451
Timing of operation	1.247	0.566	4.830	1	0.035	1.105	10.189
Segments of lesion	0.487	0.575	0.873	1	0.401	0.174	1.879

**Table 4:** The multi factor Logistic regression analysis results of the reasons for recurrent complex spinal tuberculosis accompanied with sinus tract formation.

Risk factor	Regression coefficients	Standard errors	Chi-square value	Degree of freedom	*p*-value	95% confidence interval	
						Lower	Upper
Existence of drug-resistant mycobacterium tuberculosis	2.705	0.479	22.394	1	0.000	2.874	30.951
Postoperative standard chemotherapy	1.845	0.532	20.437	1	0.016	4.201	31.203
Completeness of lesion debridement	1.632	0.347	9.962	1	0.003	1.002	8.185
Reconstruction of the spinal stability	1.374	0.359	11.694	1	0.001	1.677	10.598
Nutritional status	1.337	0.372	11.543	1	0.001	1.790	8.419
Accompaniment by tuberculosis in other organs	1.249	0.475	10.061	1	0.005	1.137	8.258
Timing of operation	1.087	0.448	8.013	1	0.004	3.812	25.493

### Operation results

No deaths were observed during either the operation or hospitalization. The mean operation time for the patients was 4.3 ± 1.3 h, and the mean volume of blood lost during the operation was 719.0 ± 317.2 ml. The incisions in 20 patients recovered by first intention; however, delayed recovery of the incision was found in the other patient. Secretions were obtained for the bacterial cultures, which ruled out mixed infection; the incision recovered by the second intention after regular clearing and dressing.

The abscesses were not obvious in 11 patients. Among these patients, 4 underwent only anterior bone graft fusion but not internal fixation after lesion debridement during the initial operation and were treated with lesion debridement again, followed by bone grafting and internal fixation. Three of the patients underwent anterior lesion debridement, bone graft fusion, and posterior internal fixation during the initial operation. The surgical findings showed that the recurrent lesions did not involve the internal fixation system; thus, the internal fixation was not removed. Four of the patients underwent anterior internal fixation during the initial operation, and the surgical findings showed that there were different degrees of pus-like tissues around the screw path; in addition, internal fixation loosening was found in 3 patients; therefore, the internal fixation and the screw path were removed, and the vertebral screw was implanted in the upper and lower normal adjacent vertebral bodies for the extended internal fixation.

For the 10 patients in whom the abscesses were obvious, the surgical findings all showed that the recurrent lesions involved the internal fixation system; therefore, the original internal fixation was removed and lesion debridement was performed. Subsequently, a bone graft fusion and a posterior internal fixation with a pedicle screw-rod system were performed. For all the patients, iliac bone autografts were used for the anterior intervertebral supporting bone implantations.

### Clinical functions and imaging assessments

Sixteen patients were healed, while recurrence was found in 5 patients, yielding a second recurrence rate of 23.8% (5/21). The 9 patients accompanied by neurological dysfunctions all recovered, to different degrees, by postoperative year 2, and the ASIA degrees were D and E in 3 and 6 patients, respectively (Table 5). The imaging examinations confirmed bone graft union at postoperative month 6 in 15 patients, at postoperative month 9 in 4 patients, and at postoperative year 1 in 2 patients. The mean Cobb angle of the kyphosis was 8.5 ± 1.9° after the operation.

**Table 5:** Neurological functional recovery according to the ASIA grade.

Preoperative	Cases	Postoperative				
		A	B	C	D	E
A	0	0	0	0	0	0
B	1	0	0	0	1	0
C	2	0	0	0	1	1
D	6	0	0	0	1	5
E	12	0	0	0	0	12

Intraoperative pleural injuries were found in 2 patients with tuberculosis in the thoracolumbar segment; after pleural neoplasty and closed thoracic drainage, the patients recovered well after the operation. Delayed recovery of the incision was found in 1 patient, whose bacterial culture of the secretion ruled out mixed infection; the incision of this patient recovered after local dressing and nutritional support. Recurrence occurred in 2 patients in whom paravertebral abscesses were not obvious before the revision operation, yielding a recurrence rate of 18.2% (2/11); we considered that the reason for the re-recurrence was the abscess and sequestrum on the contralateral side, which were not completely removed during the operation and, thus, caused the postoperative recurrence. One patient received standard chemotherapy and dressing, and the lesion recovered 3 months later; the other patient received standard chemotherapy and dressing, but CT scanning 3 months later showed that there was still an abscess on the contralateral side, and the sinus tract was not closed; thus, the anterior lesion debridement, bone graft fusion, posterior bone graft fusion and internal fixation were performed again, and subsequently, the lesion recovered. Recurrence was found in 3 patients in whom the abscesses were obvious before the revision operation, yielding a recurrence rate of 30% (3/10); the abscesses at the lesion site in all these 3 patients persisted, and reformation of the sinus tract and tuberculosis recurrence occurred. Two of the patients recovered 3 months later, following standard chemotherapy and dressing. However, in one patient, the disease did not improve after standard chemotherapy and dressing; therefore, lesion debridement was performed again, and then the lesion of the patient recovered.

## Discussion

### Reasons for the recurrence of complex tuberculosis

The results of the regression analysis showed that the postoperative recurrence of spinal tuberculosis was a process that involved multiple factors. The risk factors for the postoperative recurrence of complex spinal tuberculosis accompanied by sinus tract formation included the following:

1. Appearance of drug-resistant mycobacterium tuberculosis: Currently, the appearance of drug-resistant mycobacterium tuberculosis has become a new challenge for the treatment of tuberculosis^15,16^. In this study, 15 patients were infected with drug-resistant mycobacterium tuberculosis; although anterior lesion debridement was performed in the initial operation, the postoperative chemotherapy was non-standard or the compliance of the patients was poor, which resulted in the recurrence of tuberculosis.
2. Incomplete lesion removal: Complex spinal tuberculosis generally involves multiple vertebral bodies; in addition, this disease is commonly accompanied by tuberculosis in other organs; therefore, complete lesion removal is very difficult to achieve in such patients. In this study, all the patients had obvious residual sequestra, which were mainly found on the contralateral side of the route of the lesion debridement.
3. Failure of spinal stability reconstruction: There are still debates regarding the use of titanium meshes in anterior reconstruction of the spine in tuberculosis patients. Most researchers suggest that the application of titanium mesh is safe and effective and that it could not adversely affect the control of infection or the rate of tuberculosis recurrence^17–23^. The reconstruction of spinal stability is especially important for the treatment of tuberculosis. The positive effects of strong internal fixation and fusion on infection control and the prevention of tuberculosis recurrence have already been acknowledged by most researchers^24–26^. In this study, unfused bone grafts and the formation of pseudoarticulation was found in 5 patients, and progressive kyphotic deformities were found in 2 patients, suggesting that bony union was not achieved in these patients. The failure of the spinal stability reconstruction ultimately induced the recurrence of tuberculosis.
4. Inappropriate operation timing: ERS is an important indicator for the evaluation of operation timing in tuberculosis patients. To date, most studies have suggested that a preoperative ERS <40 mm/h is a relatively safe level. In this study, the mean ESR of the 21 patients was 67.6 ± 9.2 mm/h (~55–83 mm/h) before the initial operation; however, the symptoms of tuberculosis poisoning in the patients with complex spinal tuberculosis were relatively evident, thus only considering the control of ERS might result in missing the best operation timing, and even cause un-recovery in some paraplegic patients. Therefore, more studies are still needed to investigate the best operation timing in patients with complex spinal tuberculosis.
5. Effects of tuberculosis on other regions: Complex spinal tuberculosis is generally accompanied by tuberculosis in other organs. In this study, 6 patients were found to have tuberculosis in other organs, among whom the tuberculosis in other organs was not effectively or completely treated, which could have affected the effectiveness of the operation to a certain degree.

In addition to these risk factors, the nutritional status of the patients before and after the operations, and the standardization degree of the postoperative chemotherapy are also important risk factors. In this study, all the patients had complex spinal tuberculosis, and postoperative recurrence occurred in all these patients. The disease course in these patients was relatively long; in addition, the patients exhibited sinus tract formation, relatively heavy exudation, low haemoglobin and plasma total protein levels, and poor nutritional status. Therefore, small amounts of fresh erythrocyte suspension and human albumin were infused multiple times to correct the malnutrition and to improve the immunity of the patients.

### Treatment method and clinical prognosis of the recurrent patients with sinus tract formation

The accompaniment by sinus tract formation after operation of spinal tuberculosis is a relatively common and severe pathological type of the disease, which is mainly caused by the postoperative recurrence or ruptures of abscesses and has been considered a major challenge in clinical practice. If not treated timely and effectively, such lesions could greatly affect the life quality of the patients. Extended sinus tract lesion debridement is conventionally performed in patients during the first operation; however, only very few studies have reported treatment methods for postoperative recurrent patients accompanied by sinus tract formation.

In this study, consecutive bacterial cultures were performed using sinus tract secretions for the 21 patients with complex spinal tuberculosis accompanied by sinus tract formation to rule out mixed infection, and then extended sinus tract lesion debridement, as well as anterior or posterior bone graft fusion and internal fixation, was performed for the treatment. We speculated that, after mixed infection of the sinus tract was excluded, sinus tract and tuberculous lesion removal, as well as bone graft infusion and internal fixation, could be performed in a one-stage operation as in cases of simple spinal tuberculosis. No mixed infections of the incisions were found after the operations in this study. The rate of re-recurrence was 23.8% (5/21), among which the recurrence rate of the patients with low amounts of abscesses was 18.2% (2/11) and in patients with high amounts of abscesses was 30% (3/10). The difference between these two types of patients could be associated with the fact that the diseases of the latter ones were more severe, and the abscesses were more distinctive. One of the major causes of re-recurrence is associated with the incomplete removal of the lesion.

### Safety of using internal fixation in patients with recurrent spinal tuberculosis accompanied by sinus tract formation

In addition to lesion debridement, all the patients in this study underwent internal fixation to restore the spinal stability. Restoring the stability at the local site of the lesion is of great importance for the prevention of postoperative recurrence of tuberculosis. The conventional method for restoring spinal stability is immobilization with plaster; however, this method cannot achieve complete immobilization, and the patients are generally in pain during this method. Thus, the risk of postoperative recurrence is relatively high with this method.

In recent years, a growing number of studies have demonstrated the safety profiles and effectiveness of using internal fixation in the treatment of tuberculosis^25,27^. This method could allow patients to conduct early ambulation; in addition, with respect to immobilization with plaster, internal fixation could provide more reliable local stability, which could greatly improve bone graft fusion, help correct deformities, and maintain the normal sequences in the spine. In this study, one-stage sinus tract resection, lesion debridement, and anterior bone graft fusion and anterior internal fixation were performed for 9 patients with relatively less abscesses (among which four patients only underwent anterior bone graft fusions), which achieved satisfactory treatment effectiveness after the operation. Re-recurrence was found in only 2 patients due to incomplete lesion debridement, suggesting that the paravertebral abscess was not obvious in the patients with recurrent spinal tuberculosis; anterior internal fixation is safe and effective.

However, some fundamental studies^28^ also demonstrated that bacterial clones on the surface of implanted materials could generate a polysaccharide/protein membrane in tuberculous lesions, which could help in the avoidance of the defence reactions of the host and attack from the drugs, thereby causing continuous infection by biological materials. Therefore, in this study, for the patients with a relatively high volume of abscesses, the conventional method was implemented. Although many studies have already demonstrated the safety profiles of anterior internal fixation in the treatment of tuberculosis, there are still some studies that investigated the method of anterior sinus tract resection, lesion debridement, bone graft fusion, and posterior bone graft fusion and internal fixation. The advantage of this method is that the internal fixation and lesion areas are relatively isolated; therefore, this method could theoretically reduce the risk of implantation infection.

In summary, internal fixation could effectively restore spinal stability, promote bone graft fusion, and accelerate the recovery from tuberculosis, under the premise of complete lesion debridement. In this study, the recurrences in 5 patients were associated with incomplete lesion debridement. For patients with recurrent tuberculosis, we should not just consider the appearance of the incision; selecting the appropriate operation route and achieving complete lesion debridement are critical factors for a successful operation. We suggest that, during lesion debridement, if there are obvious sequestra and abscesses on the bilateral sides, the anterior bilateral route is a very necessary route for operation.

### Advantages and precautions of sinus tract resection and lesion debridement

Previously, conventional treatments or simple sinus tract scraping were generally used for tuberculosis patients with sinus tract formation, which involves high recurrence rates and for which a secondary operation is commonly needed. In this study, aggressive surgical interventions were performed, and sinus tract resection, lesion debridement, bone graft fusion and internal fixation were performed after the general conditions of the patients were stabilized. Effective anti-tuberculosis therapy was performed after the operation, according to the results of the drug sensitivity tests, significantly reducing the rate of re-recurrence. In this study, re-recurrence was found in only 5 patients, among whom 3 recovered after strict conventional treatment, and 2 recovered after re-operation. One thing that needs to be noted is that, for the patients with postoperative recurrence combined with sinus tract formation, mixed infection with other bacteria should be ruled out before the operation, and malnutrition status should be corrected timely. In addition, tuberculosis in other organs should also be detected during the treatment for spinal tuberculosis.

### Postoperative drug therapy for patients with drug-resistant mycobacterium tuberculosis

Treatment of drug-resistant tuberculosis is a challenge all over the world^10–12,15^, as it is mainly caused by non-standard treatment and poor compliance among the patients. In this study, the mycobacterium tuberculosis cultures showed that 15 patients were infected with multi-drug-resistant mycobacterium tuberculosis; the patients did not receive the standard anti-tuberculosis treatments according to the prescriptions after the initial operations, which further increased the difficulty in treating the cases of recurrent spinal tuberculosis. In our experience, drug resistance tests for mycobacterium tuberculosis should be performed again after the revision operation; an appropriate chemotherapy strategy should be decided according to the results of drug sensitivity tests and drug resistance tests, and the treatment duration should be 18–24 months. In addition to appropriately choosing the first- and second- line anti-tuberculosis drugs, quinolones, isoniazide, and amikacin should also be used in combination to enhance the effectiveness of anti-tuberculosis treatments. Furthermore, chemical treatment management should be performed throughout the anti-tuberculosis treatment of the patients, which is of great importance for the treatment of drug-resistant mycobacterium tuberculosis^29,30^.

### Limitations of the present study

In this study, 21 patients who were accompanied by sinus tract formation were included, preoperative cultures of the secretions, as well as cultures of the tissues from different segments during the operations, showed no signs of mixed infection with other bacteria. This finding could have been associated with the fact that once the sinus tract was formed, regular cleaning and dressing of the sinus tract were performed. In addition, all the patients in this study were treated with spinal tuberculosis operations; the continuous use of anti-tuberculosis drugs, some of which broad-spectrum antibiotics, could result, to a certain degrees, in the elimination of the effects against common bacteria, which therefore could have affected the results of the bacterial cultures in this study. Therefore, we could not clarify the incidence of mixed infections in patients accompanied by sinus tract formation after the operation.

In summary, the clinical treatment for complex recurrent spinal tuberculosis accompanied by sinus tract formation is very difficult. The major causes of recurrence included the appearance of drug-resistant strains, non-standard postoperative chemotherapy, incomplete lesion debridement, failure of spinal stability reconstruction, poor nutritional status, accompaniment by tuberculosis in other organs, and inappropriate timing of the operation. We should improve our understanding of spinal tuberculosis and treatment methods and perform aggressive anti-tuberculosis treatment against drug-resistant mycobacterium tuberculosis. Effective chemotherapy, complete lesion debridement, and appropriate spinal stability reconstruction are key factors for successful treatment.
